# HIV-1 inhibition in cells with CXCR4 mutant genome created by CRISPR-Cas9 and *piggyBac* recombinant technologies

**DOI:** 10.1038/s41598-018-26894-4

**Published:** 2018-06-05

**Authors:** Shuai Liu, Qiankun Wang, Xiao Yu, Yilin Li, Yandan Guo, Zhepeng Liu, Fuyun Sun, Wei Hou, Chunmei Li, Li Wu, Deyin Guo, Shuliang Chen

**Affiliations:** 1 School of Basic Medical Sciences, Wuhan University, Wuhan, 430071 P.R. China; 2 Institute of health inspection and testing, Hubei Provincial Center for Disease Control and Prevention, Wuhan, 430079 P.R. China; 3 School of Medicine (Shenzhen), Sun Yat-sen University, Guangzhou, 510080 P.R. China; 4 Center for Retrovirus Research, Department of Veterinary Biosciences, The Ohio State University, Columbus, OH 43210 USA

## Abstract

The C-X-C chemokine receptor type 4 (CXCR4) is one of the major co-receptors for human immunodeficiency virus type 1 (HIV-1) entry and is considered an important therapeutic target. However, its function in maintaining the development of hematopoietic stem cells (HSC) makes it difficult to be used for HIV-1 gene therapy with HSC transplantation. A previous report showed that the natural CXCR4 P191A mutant inhibits HIV-1 infection without any defect in HSC differentiation, which could provide a basis for the development of new approaches for HIV-1 gene therapy. In the present study, we used CRISPR-Cas9 combined with the *piggyBac* transposon technologies to efficiently induce the expression of the CXCR4 P191A mutant in an HIV-1 reporter cell line, leading to no detectable exogenous sequences. In addition, no off-target effects were detected in the genome-edited cells. The decline of HIV-1 replication in biallelic *CXCR4* gene-edited cells suggests that individuals equipped with homologous recombination of the CXCR4 P191A mutant could prevent or reduce HIV-1 infection. This study provides an effective approach to create a CXCR4 mutation with HIV-1 infection inhibition function and without leaving any genetic footprint inside cells, thereby shedding light on an application in HIV-1 gene therapy and avoiding side effects caused by deficiency or destruction of CXCR4 function.

## Introduction

Human immunodeficiency virus type 1 (HIV-1) can utilize the primary cellular receptor CD4 and co-receptor C-C chemokine receptor 5 (CCR5) or C-X-C chemokine receptor type 4 (CXCR4) to enter into cells by membrane fusion^1^. The CCR5 co-receptor is predominantly utilized by the R5-tropic strains of HIV-1 when a new infection is established. If the infection continues to a later stage, R5-tropic HIV-1 can change into a dual-tropism strain equipped with both R5- and X4-tropism. Emergent X4-tropism virus infection gradually becomes dominant, and CXCR4 is then utilized as an alternative receptor for HIV-1 entry. Moreover, gradually dominant virus invasion in the late stage leads to the radical loss of CD4^+^ T cells and rapid disease progression^2–5^. For HIV-1 patient treatment, in addition to the highly active antiretroviral therapy (HAART) that effectively blocks HIV-1 replication, CCR5 and CXCR4 disruption by genome engineering technologies is considered a potential strategy to inhibit viral infection^6,7^. Evidence of CCR5 as a therapeutic target was based on the finding that individuals with a high-risk for infection continued to live free of HIV-1 because of a CCR5 32bp-deletion (CCR5-Δ32) in homozygotes^8,9^. Further in clinical research, an American patient with acute myeloid leukemia and HIV-1 infection received an allogenic hematopoietic CCR5Δ32/Δ32 stem cell transplant, which not only suspended from the disease progression, but also conferred resistance to HIV infection^10,11^. However, CXCR4-based HIV-1 gene therapy has been neglected because CXCR4 deficiency led to embryonic lethality in mice and could trigger potential malignant disorders^12,13^. In addition, CXCR4 has been identified to play a critical role in maintaining normal physical function of hematopoietic stem cells^14,15^. Therefore, most studies related to CXCR4-based HIV-1 gene therapy have only been performed using primary CD4^+^T cells *in vitro*, and have not yet advanced to *in vivo* or clinical studies due to uncertain reliability and effectiveness^16–18^.

Our previous study confirmed that single-guide RNA (sgRNA)-Cas9-mediated disruption of CXCR4 in primary CD4^+^T cells can efficiently block HIV-1 infection^19^. However, the nucleotide indels induced from homologous recombination (HR) or non-homologous end joining (NHEJ) after DNA double-stranded breaks (DSBs) may impair the original functions of CXCR4 *in vivo* if applied to clinical HSC transplantation^20^. A previous report showed that a natural CXCR4 mutant (P191A) can abrogate its binding to the HIV-1 envelope protein gp120 without affecting its normal function *in vivo*^21^. These discoveries prompted us to find a way to replace wild-type CXCR4 with the CXCR4 (P191A) mutant, to confer resistance against HIV-1 infection without affecting normal immune functions in HSC development. We have reported that silencing CXCR4 expression with shRNA and introducing a CXCR4 (P191A) mutant with a lentiviral vector inhibits HIV-1 replication by approximately 60% without impairing CXCR4-mediated downstream signal transduction^22^. However, this approach allowed for extra insertion of lentiviral vector in genome, which may cause tumorigenesis as a side effect. In order to overcome this caveat, we combined CRISPR-Cas9 with a *piggyBac* transposon system to construct a natural CXCR4 (P191A) mutant without any redundant DNA in the genome. The *piggyBac* transposon system, as an alternative non-viral mobile genetic element, was found to be more efficient in mediating nucleotide sequence transposition in host cells and more promising for human gene therapy^23–25^.

Most corrections of genetic disorders or creation of genetic mutations in mouse models are achievable by homologous recombination (HR)^26^. However, gene targeting by template-mediated zinc finger nucleases or CRISPR-Cas9 technology results in relatively low HR, and the efficiency can be increased by introducing DSBs^27,28^. In order to improve gene targeting and efficiency of HR induced by DSBs, we explored the most efficient sgRNA-Cas9 for *CXCR4* genome cleavage with the two targeted sequences close to the site of the CXCR4 (P191A) mutation^19,29^. In addition, we achieved HR by a *piggyBac*-based transposon system with a drug selection marker to enhance HR efficiency^24,30^. By combining a CRISPR-Cas9 strategy and *piggyBac* transposon system, we achieved efficient replacement of wild-type CXCR4 with a CXCR4 P191A homozygous mutant in an HIV-1 reporter cell line and observed significant inhibition of HIV-1 infection. Through this study, we established an efficient strategy to introduce CXCR4 P191A mutation, which may eventually be used for clinical HIV-1 gene therapy research. Our approach also provided a potential gene correction method for treatment of genetic disorders in the future.

## Results

### Construction of CXCR4 mutant (P191A) targeting vectors using CRISPR-Cas9 and *piggyBac* transposon system

**Figure 1:**
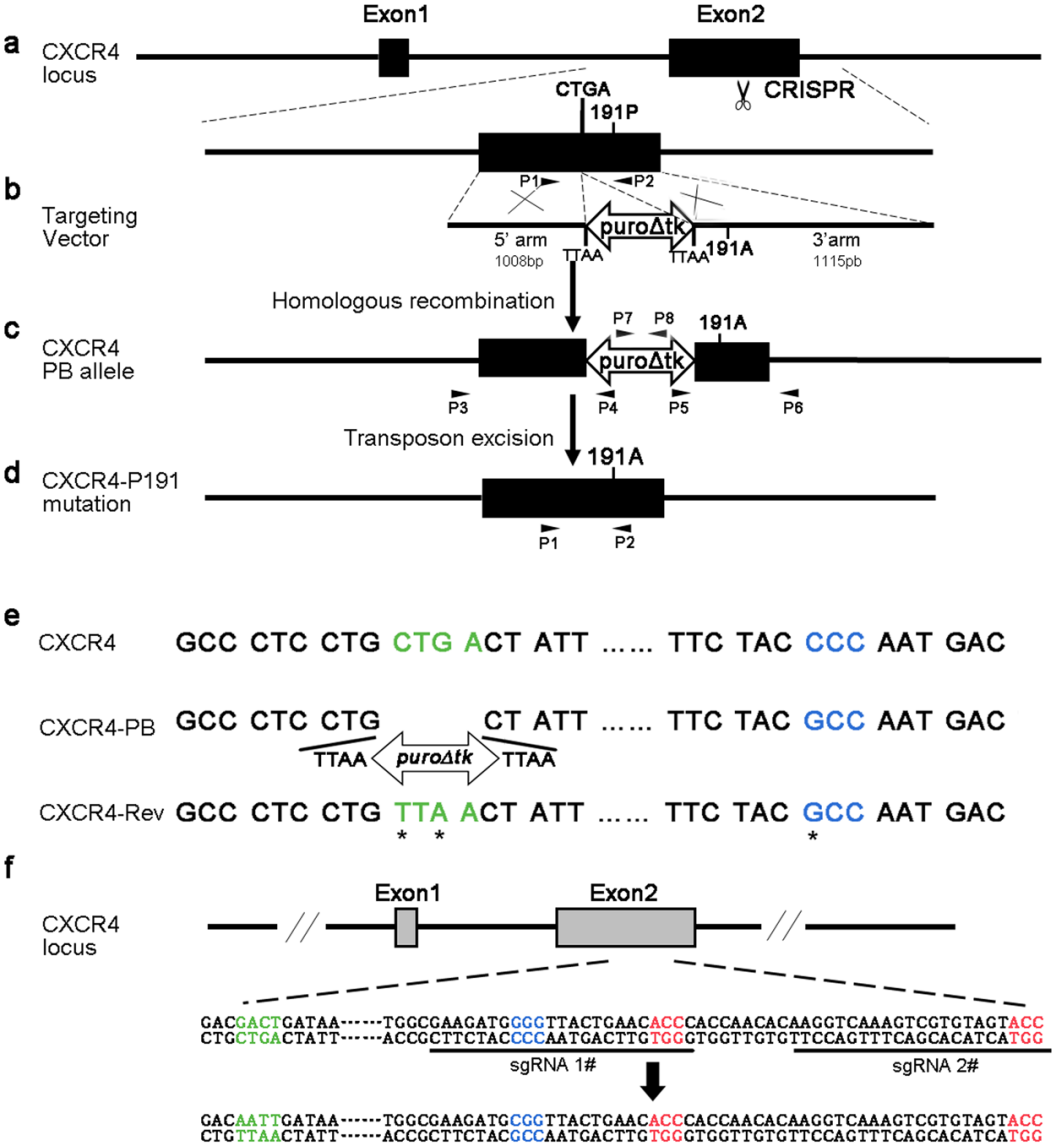
Schematic diagram of the genomic edition and HR steps to generate CXCR4-P191A mutation using CRISPR/Cas9 and *piggyBac* transposon. (**a**) Structure of the *CXCR4* gene and CRISPR/Cas9 cleavage position on the *CXCR4* exon 2. (**b**) The targeting construct of the *piggyBac* transposon carrying the selectable markers, puroΔtk, flanked by about 1 kb of genomic sequences from wildtype and point mutations that introduced alanine substitution of residue P191. (**c**) Insertion of the *piggyBac* cassette following HR. After selection with puromycin, clones with HR were identified by PCR amplification of 5′arm with P3 and P4, 3′arm with P5 and P6, and *CXCR4* genome with P1 and P2. (**d**) The seamless CXCR4 P191A mutation-corrected clones were isolated by transfection with a transposase-expressing plasmid. (**e**) Sequences of wild-type (CXCR4), PB and Rev alleles. Amino acid position 191 (blue), *piggyBac* excision site (green) are shown. Sequence changes in CXCR4-Rev allele were indicated by asterisks. (**f**) Schematic of the sgRNAs for targeting exon2 of wild-type *CXCR4* gene. Amino acid position 191 (blue), recognition sites for Cas9 (underline) and *piggyBac* excision site (green) are shown.

To introduce the naturally occurring human CXCR4 mutant P191A, we developed a strategy to precisely generate the P191A mutation without leaving any redundant sequences in the targeted genome. Upon examination of the sequences 69-bp upstream of the P191A mutation, we found that replacing the leucine codon sequence CTG with the leucine codon TTA allows the *piggyBac* transposon cassette be excised and released from the host genome^31^ without affecting the amino acid sequence of CXCR4 (Fig. 1a). The leucine codon CTG is located upstream of the targeted P191A mutation site. The original donor vector contains two inverted terminal repeat sequences of the *piggyBac* transposon and a bi-functional hybrid *puroΔtk* gene for positive and negative selection^32^ (Fig. S1a). We designed a targeting vector carrying the homologous sequence with the 191A mutation in the 3′ arm region and a *PGK-puroΔtk* cassette flanked by *piggyBac* repeats inserted into the TTAA sites (Fig. 1b). The homologous sequence on the 5′ arm ends up with the CTG sequence and the 3′ arm started downstream of the CTG codon (Fig. 1b). The *piggyBac* cassette can be inserted precisely at this CTG site and replaced with TTA through HR, thus allowing selection of the genome-edited cells with puromycin (Fig. 1c,e). After transfection with transposase plasmid into TZM-bl cells, the *piggyBac* transposon containing the selection cassette inserted at the TTAA site can then be excised from the genome, leaving the CXCR4 P191A mutation behind (Fig. 1d,e). Further cell sorting by flow cytometry with anti-CXCR4 specific antibody can distinguish the cells with CXCR4 mutant P191A from the cells that retain the *piggyBac* transposon cassette carrying the *tk* gene. To induce DSBs in the region of CXCR4 P191A loci, we designed two sgRNAs to induce Cas9 precisely targeting the DNA adjacent to both the CGTA sequence and the P191A loci. Each sgRNA has 20-bp guide sequences adjacent to a protospacer adjacent motif (PAM) domain containing the canonical trinucleotide NGG to target the *CXCR4* gene (Fig. 1f).

### CRISPR/Cas9-mediated gene targeting of the *CXCR4* loci and site-specific HR in TZM-bl cells

**Figure 2:**
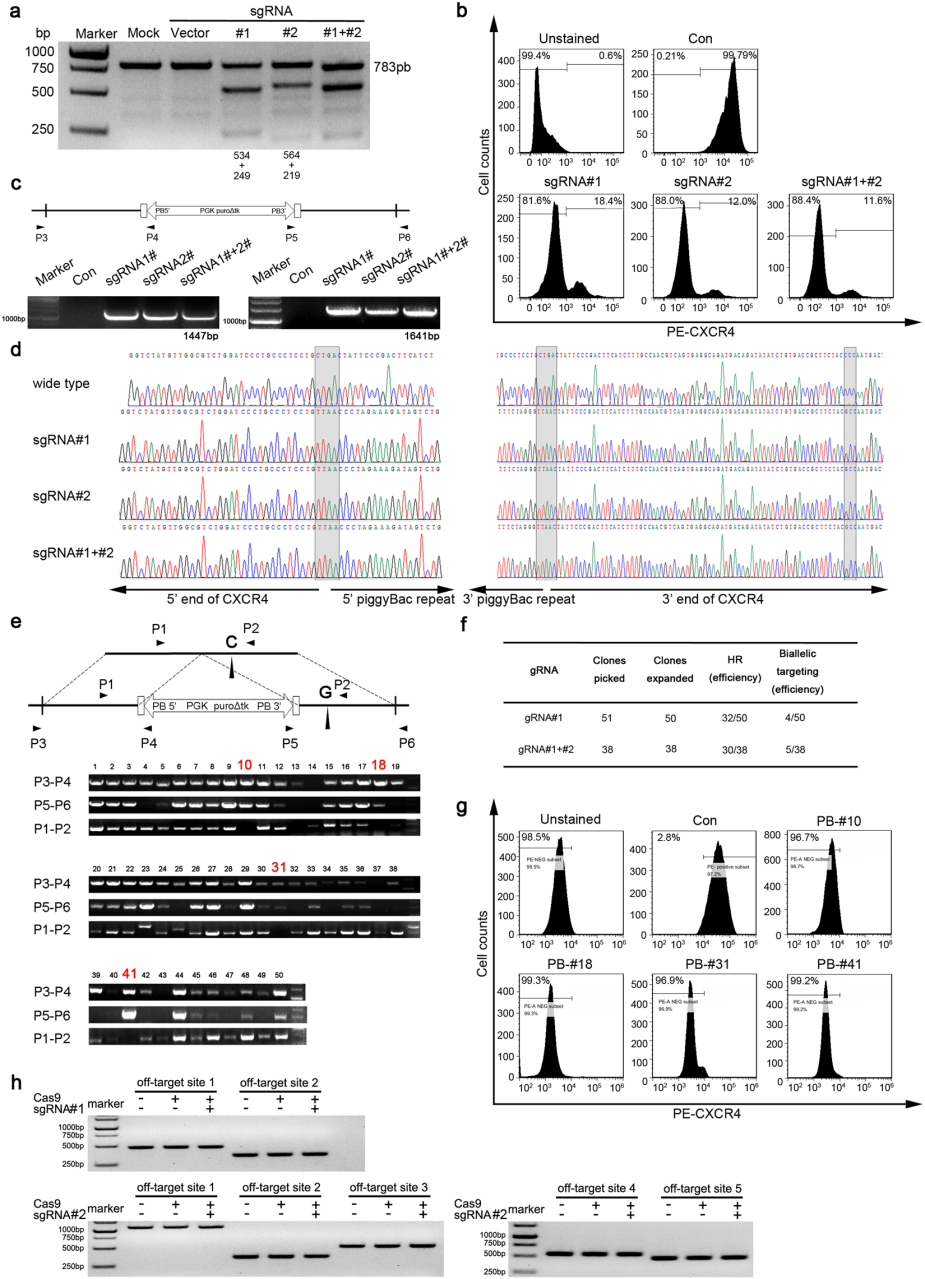
CRISPR/Cas9-mediated gene targeting of the *CXCR4* loci and site-specific HR in TZM-bl cells. (**a**) T7E1 assay of designed sgRNA#1 and sgRNA#2 induced lentiCRISPR/Cas9 targeting CXCR4 in TZM-bl cells. TZM-bl cells (5.0 × 10^5^) were transfected with sgRNA-lentiCRISPR/Cas9 plasmids as indicated. After 72 h, the genomic DNA from the cells was extracted and PCR amplified to test for CXCR4 disruption by T7 endonuclease I assay using a 1.5% agarose gel. The lower migrating bands in lanes indicate the disrupted *CXCR4* alleles. (**b**) Flow cytometry analysis of surface CXCR4 expression in lentiCRISPR/Cas9-transfected TZM-bl cells. The TZM-bl cells (5.0 × 10^5^) transfected with CXCR4-sgRNA#1-, sgRNA#2- or sgRNA#1 and #2-lentiCRISPR/Cas9 plasmids were stained with PE-conjugated antibody specific for CXCR4 and analyzed by flow cytometry. The stained TZM-bl cells were used as a positive control (Con). The values shown are the percentages of CXCR4 positive or negative cells. (**c**) (**d**) PCR identification and Sanger sequencing analysis of CRISPR/Cas9 mediated site-specific HR in TZM-bl cells. The TZM-bl cells were transfected with sgRNA#1-, sgRNA#2-, or sgRNA#1 and #2-lentiCRISPR/Cas9 together with transposon *piggybac* donor vector plasmids. After 14 days selection with puromycin, two pairs of PCR primers, P3/P4 and P5/P6 were used to detect integration events. Sanger sequencing was used to confirm the correct integration. (**e**) Cell clones with correct biallelic targeting were selected and further identified by PCR amplification. The upper and middle lanes indicate the PCR gel results of CRISPR/Cas9 targeted cell clones, while the bottom lane shows biallelic targeting clones. (**f**) Summary of successful specific HR in CRISPR/Cas9-mediated gene targeting. The expanded clones represent successfully passaged clones. (**g**) Flow cytometry analysis showing the lack of CXCR4 expression in screened TZM-bl cells. (**h**) Off-target analysis for Cas9/sgRNA#1 and Cas9/sgRNA#2-mediated targeting. Seven potential off-target sites were PCR-amplified from Cas9/sgRNA#1 and Cas9/sgRNA#2-treated cells and mock cells. These amplicons were subjected to T7E1 assay to reveal any off target sites between the targeted cells and mock cells by gel electrophoresis.

To test the specificity and efficiency of customized sgRNA#1 and sgRNA#2 to induce Cas9-mediated DSBs in the *CXCR4* gene, CRISPR/Cas9-sgRNA and a targeting vector containing the *piggyBac* cassette (Fig. 1b) were co-transfected into TZM-bl cells, which are derived from human HeLa cells that have high expression of CXCR4^33^. Three days after transfection, we measured the efficiency of insertion/deletions at the *CXCR4* loci using a T7E1 assay. The results showed that the designed CRISPR/Cas9-sgRNA system was able to cleave the CXCR4 genome and induce DSBs at the targeted site (Fig. 2a). In a parallel experiment, we used a flow cytometry assay to analyze cell surface CXCR4 protein levels three days post-transfection. The results showed that the population of CXCR4-positive cells was reduced from 99.8% to 18.4% and 12.0% after treatment with sgRNA#1 and sgRNA#2 respectively, and to 11.6% after treatment with the combination of both sgRNA#1 and sgRNA#2 (Fig. 2b), In order to confirm site-specific HR, we selected the transfected cells for two weeks with puromycin and then examined the genome by PCR analysis and Sanger sequencing. As we expected, the puromycin-resistant cells obtained the *piggyBac* cassette, which was detectable by PCR (Fig. 2c). In addition, the DNA sequencing results revealed that the CRISPR/Cas9-sgRNA system induced site-specific HR in the targeted CXCR4 loci and introduced the P191A mutation (Fig. 2d). In order to analyze HR efficiency, 50 single cell clones were picked and further identified by PCR (Fig. 2e). We noticed that HR induction by CXCR4-sgRNA#1 and the *piggyBac* transposon occurred in 64% (32/50) of cells, of which 8.0% (4/50) were biallelic mutations (Fig. 2f). In addition, we observed HR induction by two CXCR4-sgRNAs in 78.9% (30/38) of cells, of which 13.2% (5/38) were biallelic mutations (Fig. 2f). This suggests that multi-sgRNAs result in higher HR efficiency and more biallelic targeting compared to individual sgRNA. Moreover, we propagated four biallelic targeting clones (#10, #18, #31 and #41) to analyze CXCR4 expression by flow cytometry. The results showed that all selected single cell clones had homogeneity without CXCR4 expression (Fig. 2g).

To test the specificity of the lentiCXCR4-sgRNA/Cas9 and *piggyBac* transposon system in gene targeting, the predicted sgRNA off-target sites were analyzed. Two and five potential off-target sequences with high scores for sgRNA#1 and sgRNA#2, respectively, were identified and PCR amplified for T7E1 assay. Off-target mutations were not detectable in lentiCXCR4-gRNAs/Cas9 and *piggyBac* transposon transfected cells (Fig. 2h), suggesting high specificity of the gene targeting system.

### Generation of the CXCR4 mutant P191A by *piggyBac* transposase excision in TZM-bl cells

**Figure 3:**
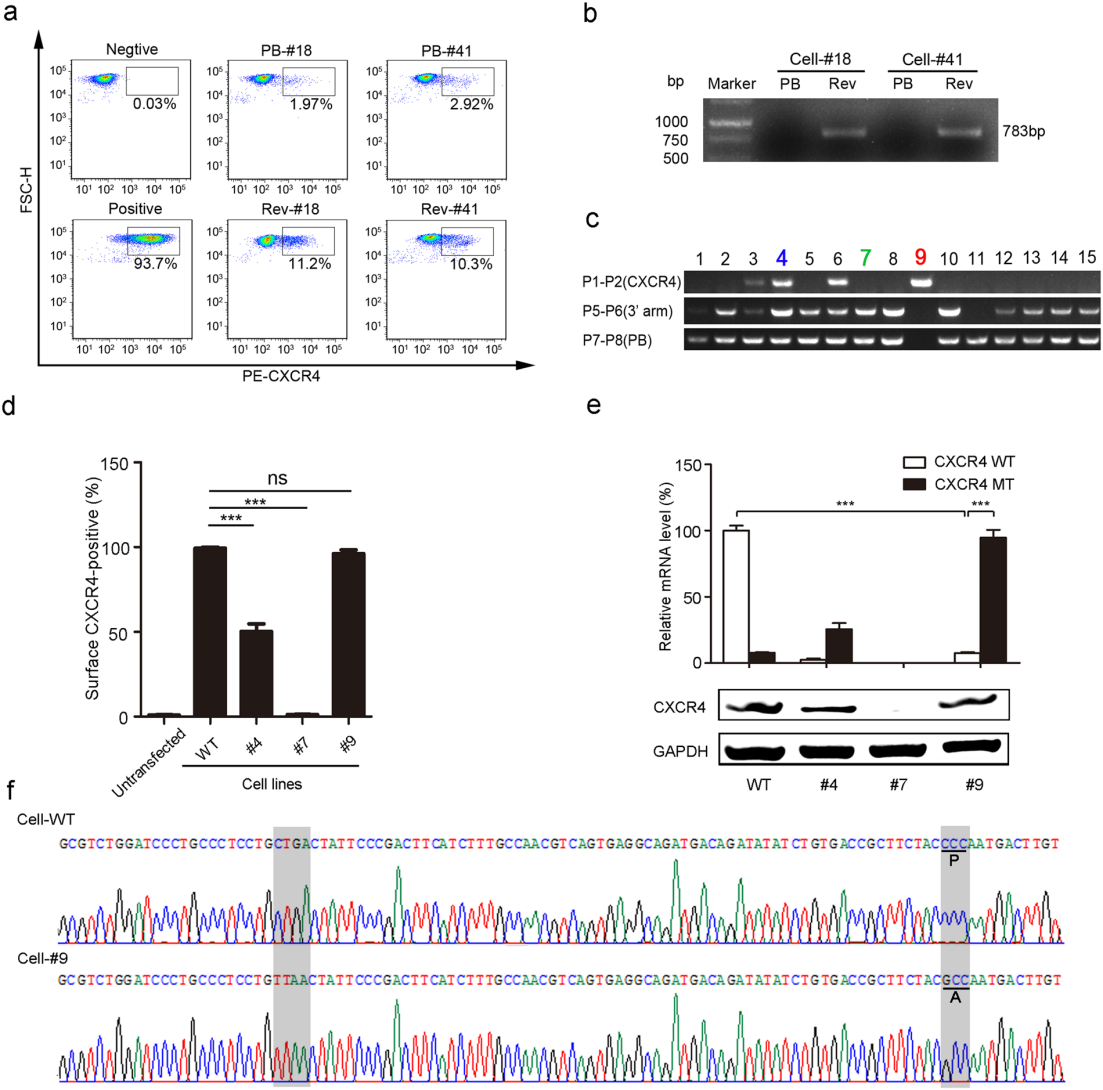
Generation of the seamless CXCR4 mutant P191A by *piggyBac* transposase excision in the HR TZM-bl cells. (**a**)The biallelic targeting cells transfected with transposase expression plasmids were stained with PE-conjugated antibody specific for CXCR4 and analyzed by flow cytometry. PB: the cells with successful specific HR. Rev: the cells with re-integrated genome containing CXCR4-P191A variant. (**b**) PCR analyses using a pair of CXCR4 primers. (**c**) Cell clones were analyzed after transposon excision using PCR amplification. (Top) Presence of the CXCR4 band indicates successful generation of the CXCR4 intact genome using P1 and P2 primers. (Middle) Absence of a band indicates biallelic excision of the transposon at the CXCR4 locus using P5 and P6 primers. (Bottom) Absence of a band indicates complete removal of transposon from the genome, using P7 and P8 primers. (**d**) Percentage of surface PE-CXCR4 in screened and verified cell lines. Results were quantified and compared to wild-type. Mean ± SD represent the average value of three experiments. ***P < 0.001, ns. indicates not significant. (**e**) Real-time qPCR was performed for detection of CXCR4 mRNA levels and Western blot analysis of CXCR4 expression in wild-type and mutant cell line. Data were normalized to wild-type CXCR4 mRNA level in non-transfected cells and expressed as relative mRNA level. Mean ± SD of three independent experiments are shown. (**f**) Sequence analysis showing the junction and the genomic sequences before transposon removal (CXCR4-wild-type) and the restoration of the sequence after transposon removal (CXCR4-P191A). The TTAA sequence of the *piggyBac* that is used for insertion and excision from the genome.

To remove the *piggyBac* transposon cassettes from the targeted genome and generate an intact *CXCR4* gene with the P191A mutation, we transfected two homozygously targeted TZM-b1 cell clones (Cell #18 and Cell #41) with a plasmid encoding hyperactive *piggyBac* transposase (pCMV-HyPBase). Seven days after transfection, flow cytometry was performed to analyze CXCR4 expression and PCR was used to detect the CXCR4 genome. We found that the number of cells with CXCR4 (P191A) expression increased from 1.97% and 2.92% to 11.2% and 10.3% after treatment with transposase excision in cell clones #18 and #41, respectively, suggesting that the reintegration of CXCR4 mutation genome leads to re-expression of CXCR4 (Fig. 3a). In a parallel experiment, we observed PCR amplification of *CXCR4* gene fragments after *piggyBac* cassette removal by transposase (Fig. 3b). To identify single TZM-bl cell clones harboring the CXCR4 P191A mutation, clone 18# with biallelic transposon insertion was transfected with *piggyBac* transposase expression vector. After transfection, 15 new clones were picked for PCR screening with three pairs of primers designed to amplify recombinant CXCR4, the 3′ arm, and the *puroΔtk* gene (Fig. 3c). We found three distinct genotypes of TZM-bl cell clones, with clone #4 showing transposon removal from one allele, clone #7 retaining transposons in both alleles, and clone #9 showing transposon removal from both alleles (Fig. 3c). This result was further confirmed by flow cytometry analysis of CXCR4 expression (Fig. S2). Compared with wild-type cell lines, there was no reduction of surface CXCR4 expression in clone #9. However, the surface CXCR4 expression was reduced to 49.2% in clone #4 and 1.13% in clone #7 (Fig. S2). Statistical analysis of the surface CXCR4 expression in different cell lines showed that the level of CXCR4 mutant expression in clone #9 was comparable to that in the wild-type cell line (Fig. 3d). In order to test whether expressed CXCR4 in clone #9 is the P191A mutant or wild-type, we designed two pairs of primers differing by a specific tetranucleotide sequence, TTAA or CTGA, which can distinguish the CXCR4 mRNA of the mutant from wild-type (Fig. 3f). We applied qPCR to evaluate the relative mRNA expression of wild-type and mutant CXCR4. The results showed that, in reformed cell clone #9, the level of the mutant CXCR4 mRNA was much higher than residual wild-type CXCR4, and was almost the same level as wild-type CXCR4 mRNA in wildtype cells (Fig. 3e). Western blot analysis revealed that the protein expression level of CXCR4 in cell clone #9 was restored to the level of wildtype cells (Fig. 3e). Using gene sequence alignment, the seamless removal of the *piggyBac* transposon was confirmed by the reintegration of the CXCR4 without any exogenous sequence inserted into the genome (Fig. 3f). To eliminate any undesirable influence on cell growth due to screening process, we measured cell apoptosis to determine whether there was a difference in cell survival. The cell apoptosis result revealed that *CXCR4* editing did not significantly affect the cell growth of genome-edited cells compared to wild-type cells (Fig. S3).

### TZM-bl cells engineered to express CXCR4-P191A suppress HIV-1_NL4-3_ infection

**Figure 4:**
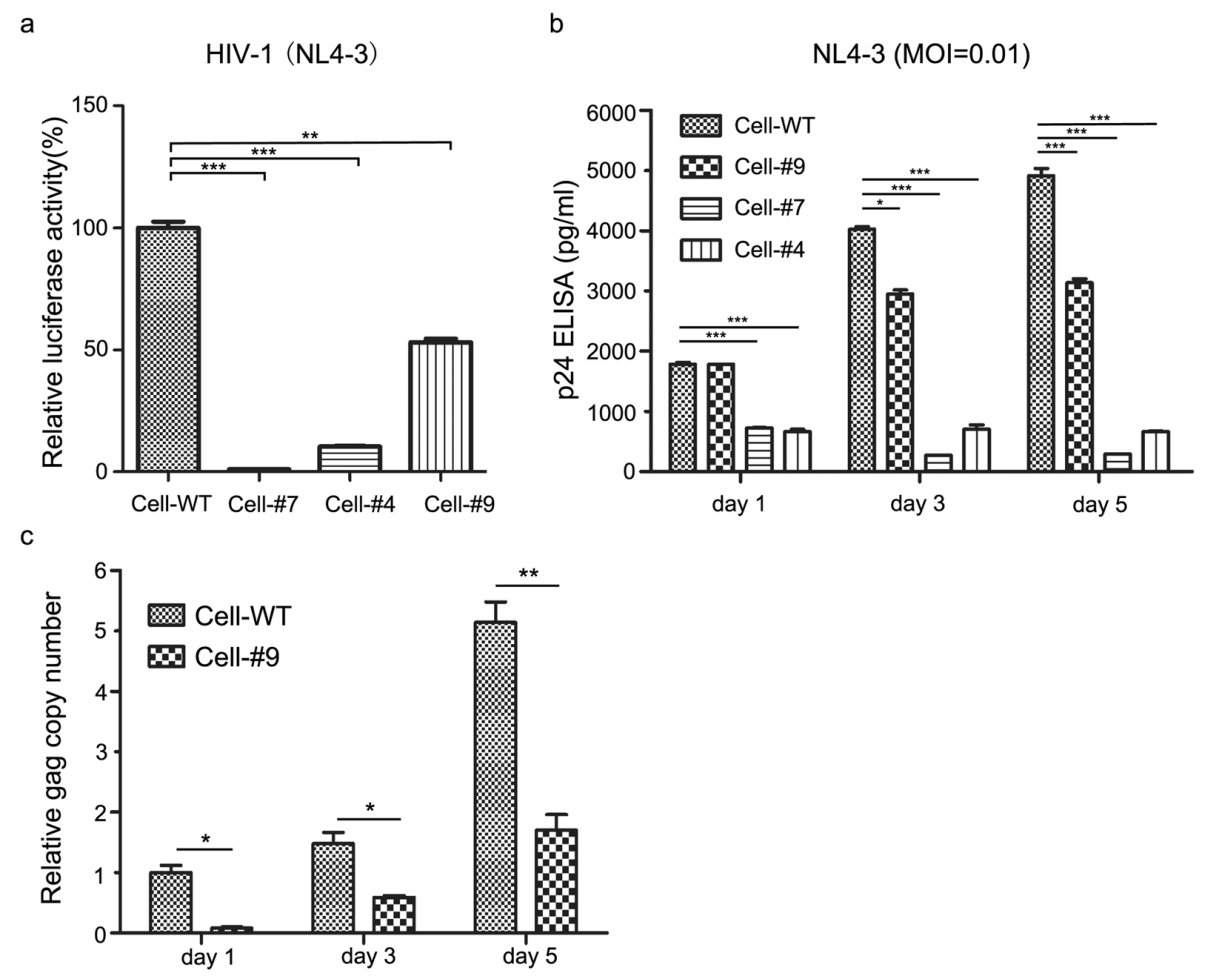
TZM-bl cells of CXCR4 mutant P191A created by CRISPR/Cas9 and *piggyBac* transposon confer significant reduction of X4-tropic HIV-1 infection. (**a**) The luciferase report assays of HIV-1_NL4-3_ infection and replication in different CXCR4 genome modified TZM-bl cells. The luciferase activities were detected 3 days post HIV-1_NL4-3_ infection. The data shown were the mean ± SD of three independent experiments and normalized to wild-type cells. Statistical significance is determined by Student’s t test. (**b**) p24 ELISA assays of HIV-1_NL4-3_ infection and replication in CXCR4 genome-edited TZM-bl cells. Different CXCR4 genome modified TZM-bl cells were challenged with HIV_NL4-3_ at a multiplicity of infection (MOI) of 0.01. The cell culture supernatants were collected on 1, 3, 5 days post-infection and p24 protein levels were measured by ELISA. The challenge experiments were performed in triplicates and repeated three times. Statistical significance was determined by Student’s t test. (**c**) qPCR detection of HIV-1_NL4-3_ replication level in wild-type and mutant TZM-bl cells. Assays were performed as in (**b**). The relative copy numbers of gag were normalized to β-globin. Data were normalized to gag gene level in wild-type cells 1 day after HIV-1_NL4-3_ infection. *P < 0.05, **P < 0.01, ***P < 0.001.

To assess the impact of the replacement of endogenous CXCR4 with mutant CXCR4 (P191A) on HIV-1 infection, we utilize a TZM-bl cell line containing a luciferase reporter driven by the HIV-1 long terminal repeat (LTR) promoter, which is used to evaluate and quantify HIV-1 infection^33^. Cells expressing the homozygous CXCR4 (P191A) mutant (cell clone #9) showed a 50% reduction in HIV-1 replication when compared to the wild-type cells; however, cell clone #4, with transposons removed from one allele, and cell clone #7, with transposons retained in both alleles, showed a significant inhibitory effect due to the destruction of CXCR4 integrality (Fig. 4a). We then tested whether the expression of CXCR4 (P191A) protected cells from HIV-1 infection. HIV-1 p24 levels in the supernatants of replication-competent HIV-1_NL4-3_ infected cells were measured at 1, 3, and 5 days post-infection. The results indicated that the level of p24 gradually increased during HIV-1 infection and the cells (clone #9) with CXCR4 P191A resulted in partial reduction compared with wild-type cells (Fig. 4b). In a parallel experiment, the cells were collected to examine the level of gag mRNA, and the qPCR result showed that the copy number of HIV-1_NL4-3_ significantly decreased 5 days post-infection in the cells expressing CXCR4 mutant P191A compared to wild-type cells (Fig. 4c). Taken together, these results demonstrated that the replacement of endogenous CXCR4 with the P191A mutant significantly reduces HIV-1_NL4-3_ infection.

### TZM-bl cells engineered to express CXCR4 mutant P191A can specifically restrict the infection of X4- tropic instead of R5-tropic HIV-1 strains

**Figure 5:**
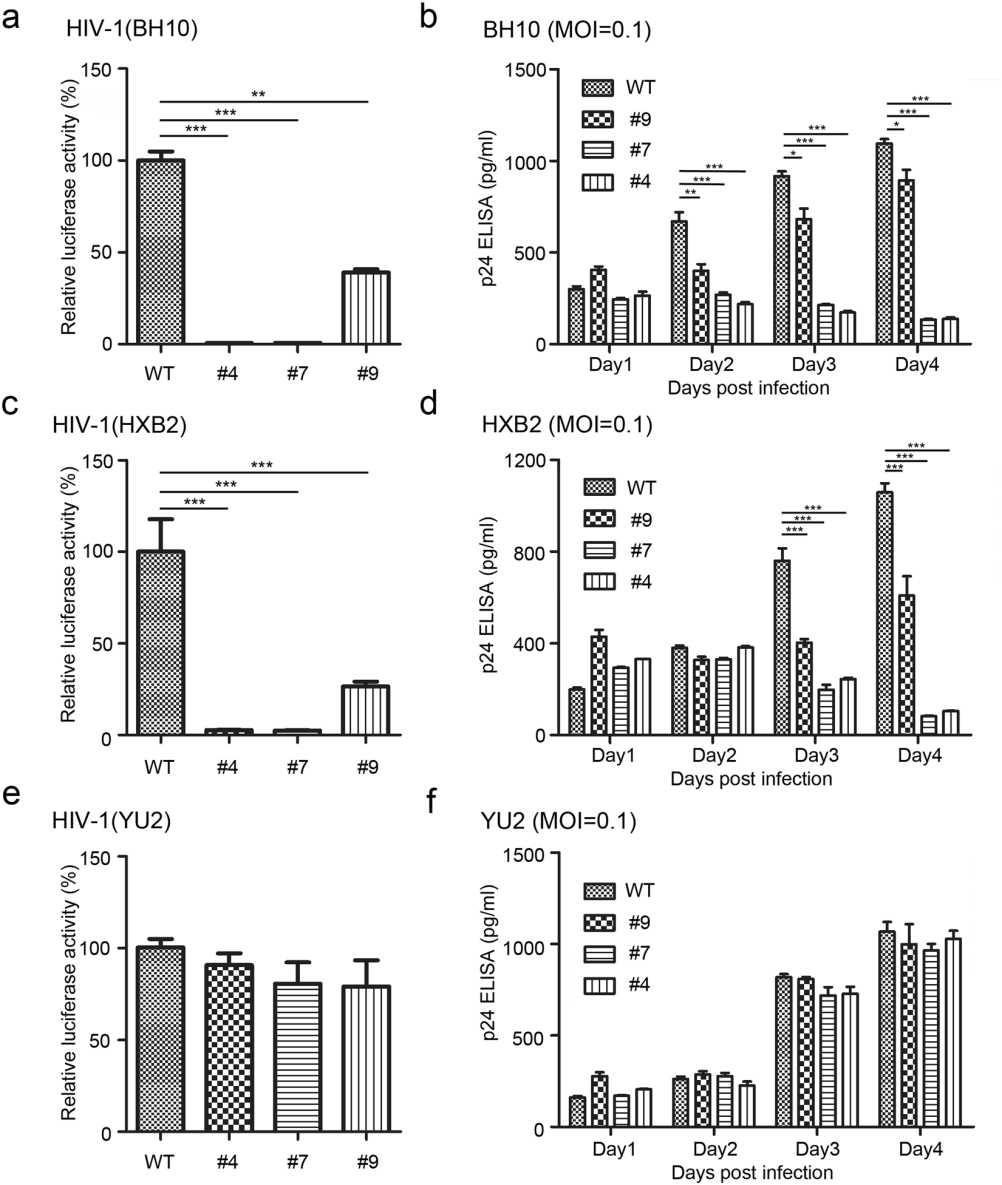
TZM-bl cells engineered to express CXCR4 mutant P191A can specifically restrict the infection of X4-tropic - but not R5-tropic HIV-1 strains. (**a**, **c** and **e**) Luciferase reporter assay to quantify relative HIV-1 infection levels in different cell lines. Screened and verified TZM-bl cell lines were infected with HIV-1 (**a**) HIV-1_BH10_ (**c**) HIV-1_HXB2_ (**e**) HIV-1_YU2_ for 8 h, then washed three times with PBS and cultured in complete DMEM medium for 48 h. Cells were collected and lysed in 100 μl of lysis buffer (Promega) for luciferase assay. The data shown are the mean ± SD of three independent experiments and normalized to wildtype cells. Statistical significance was determined by Student’s t test. (**b**, **d** and **f**) HIV-1 p24 was measured by p24 ELISA in the supernatant of different cell lines. At the indicated time points post-infection with (**b**) HIV-1_BH10_ (**d**) HIV-1_HXB2_ (**f**) HIV-1_YU2_ (MOI = 0.1), the culture supernatants were harvested for ELISA detection of HIV-1 p24 expression. Mean ± SD represents the average value of three independent experiments. *P < 0.05, **P < 0.01, ***P < 0.001.

After successfully blocking HIV-1_NL4-3_ infection, we attempted to test the effect of the CXCR4 mutant P191A on restricting infection of other X4-tropic HIV-1 strains. The HIV-1 challenge assay was performed with HIV-1_BH10_ and HIV-1_HXB2_ infection in screened and validated cell lines. Quantification of virus replication was evaluated by luciferase reporter assay at 48 h post infection. The results revealed that HIV-1_BH10_ and HIV-1_HXB2_ infection in cell-#4, cell-#7 and cell-#9 was largely decreased relative to that in the wild-type cells (Fig. 5a,c). In addition, HIV-1 infection in cell-#9 showed an approximate 50%~60% reduction compared to cell-WT (Fig. 5a,c). Furthermore, viral p24 levels in the culture supernatants were measured every day post-infection by p24 ELISA, for up to 4 days. The results indicated that the levels of p24 released from cell-#9 were significantly decreased compared to the wild-type cell lines (Fig. 5b,d). To verify whether the CXCR4 P191A mediated inhibition effect is specific to X4-tropic HIV-1 strains, we subsequently tested R5-tropic strain HIV-1_YU2_ infection in screened cells by luciferase reporter and p24 ELISA assays. The results showed there was no difference in HIV-1_YU2_ infection among the different modified cell lines (Fig. 5e,f). These results revealed that TZM-bl cells engineered to express the CXCR4 mutant P191A can specifically limit the infection of X4- tropic rather than R5-tropic HIV-1 strains.

## Discussion

In this study, we designed a novel approach using customized CRISPR-Cas9 and *piggyBac* transposon systems to alter functional sites of CXCR4 at the genome level. First, by selecting an appropriate sgRNA-Cas9 to target *CXCR4* and induce DSBs around the coding sequence of residue P191^19^, the 191A mutation carried by the *piggyBac* template could be inserted into the genome through DSB-mediated spontaneous HR. Then, following puromycin selection and sorting of CXCR4-positive cells after transposase-guided cleavage of the selection marker, we achieved functional replacement of wild-type CXCR4 with the P191A mutant. Furthermore, we observed that no residual exogenous sequences remained at the site of editing and the genome appeared undisturbed. Consequently, the genome edited TZM-b1 cells can reduce HIV-1 infection. Therefore, this approach could provide a new strategy for CXCR4-targeted therapy against HIV-1 infection.

We chose the P191A mutation as a functional target based on previous reports, in which the authors verified that alanine substitution of P191 in the second extracellular loop in CXCR4 leads to a significant reduction in HIV-1 co-receptor activity and impairs neither ligand binding nor Ca^2+^ signaling^21,34^. In addition, our group confirmed significant suppression of HIV-1_NL4-3_ infection in cells by the expression of CXCR4 P191A mutation^22^. As a classical method for *in situ* correction of a mutated gene, HR was limited due to its low efficiency^35–37^. However, in our study, the customized gRNAs in combination with CRISPR-Cas9 directly targeted the P191 coding region and generated DSBs more efficiently. Simultaneously, this site-specific DNA cleavage promoted efficient HR when the homology-directed repairing *piggyBac* templates were introduced. Remarkably, our data showed that up to 64–78% of individual cell clones were targeted on one allele, whereas 8–13% of clones had simultaneous biallelic targeting.

In previously used Cre/Lox P system-mediated gene targeting strategies, the removal of selection cassettes presumably left residual sequences in the targeted loci, which may lead to detrimental effects in cells. For instance, the Cre/Lox P system leaves a 34-bp Lox P sequence when releasing the insertion^38,39^. However, our new strategy using the *piggyBac* construct, flanked by homologous genomic sequences, could seamlessly remove the selection cassette and reintegrate the P191A mutation into the *CXCR4* gene instead of introducing of exogenous sequences in the recombinant allele. Similar to other gene targeting methods, the caveat using our present approach in TZM-bl cells is the lack of negative selection with the *puroΔtk* gene after the selection cassette is removed. However, we can use the flow cytometry analysis with positive selection to overcome this drawback. Negative selection using thymidine kinase of the hybrid *puroΔtk* gene would discriminate and eliminate cells that retained the *piggyBac*, whether it belongs to homozygotes or carriers^40^. This negative screening strategy could be widely applied in many pure ES cells but not in TZM-bl cells, which could be due to different sensitivity to the selection drug^32,41–43^. In addition, we observed approximately 10% of total genome excision efficiency by flow cytometry analysis of CXCR4 expression after cleavage of the *piggyBac* selection cassette. We further confirmed that the seamless replacement of wild-type CXCR4 with the P191A mutation caused significant inhibition of HIV-1 infection in TZM-b1 cells, and that the mRNA level of the mutant was comparable to wild-type CXCR4. Therefore, this method could be transferred to clinical use after confirmation of its efficiency and safety in HSC. Recently, combining engineered nucleases with *piggyBac* technology to generate a CCR5Δ32 deletion in induced pluripotent stem cells (iPSCs) and in monocytes or macrophages differentiated from the modified iPSCs embraces the capability of anti-HIV infection, providing a promising alternative approach for HIV/AIDS gene therapy in R5-tropic HIV-1 infection^44^. Considering the crucial physiological role of CXCR4 in humans, knockout of the *CXCR4* gene may exert undesired harmful effects^45^. Our study provided a new approach, employing substitution of wild-type CXCR4 with a functional mutant instead of destroying the integrity of the genome to inhibit X4-tropic HIV-1 infection.

In summary, the combination of CRISPR-Cas9 to edit the *CXCR4* gene at a functional site and inducing *piggyBac* transposon-mediated HR events can efficiently generate a CXCR4 P191A mutation in the genome, which confers less efficient HIV-1 infection. This approach is also adaptable to correct genetic disorder mutations at a genomic scale.

## Materials and Methods

### Plasmid construction

The lenti-CXCR4-gRNA-Cas9 vectors were constructed as described in our previous study^19^. The *piggyBac* transposon vector, pMCS-AAT-PB:*PGKpuroΔtk*, is a gift from Dr. Allen Bradley (The Wellcome Trust Sanger Institute, Cambridge, UK). The schematic diagram was showed in Fig. S1a. To generate the CXCR4 targeting vector (pMCS-CXCR4-PB:*PGKpuroΔtk*), the fragment containing 1008-bp of the 5′ homology arm that ended with CTG sequence was amplified from the genome of normal peripheral blood mononuclear cell (PBMC) using the forward primer F (5′-AACGGCGCGCCGAGAATGAGTCTTTGCAACGCCCC-3′) and the reverse primer R (5′-AGAATGCATGCGTCAATTTTACGCAGACTATCTTTCTAGGGTTAACAGGAGGGCAGGGATC-3′) followed by purification and digestion by AscI and NsiI restriction enzyme. For the 1115-bp 3′ homology arm sequence construction, the fragment starting with DNA downstream of CTG in the CXCR4 gene (CXCR4-P191A) mutation region was amplified by three steps of PCR: First, the wildtype 3′ arm sequence was amplified from PBMC using the forward primer F (5′-TAACGTACGTCACAATATGATTATCTTTCTAGGGTTAACTATTCCCGACTTCATCTTTGCCAA-3′) and the reverse primer R (5′-GCCTTAATTAATGTCAACAGAAATCAGAACATAACACTAA-3′) followed by purification and then cloned into the pGEM-T Easy vector (Promega); second, the 3′ arm sequence with P191A mutation fragment was amplified from the recombinants using the forward primer F (5′-GACCGCTTCTACGCCAATGACTTGT-3′) and the reverse primer R (5′-ACAAGTCATTGGCGTAGAAGCGGTC-3′) followed by the digestion of DpnI restriction enzyme to accomplish the site-specific mutagenesis. In the end, the 3′ arm sequence with P191A mutation followed by purification and digestion by BswiI and PacI restriction enzyme. The two sequence (5′ homology arm and 3′ homology arm with P191A mutation) were then ligated into the AscI-NsiI and BswiI-pacI clone sites of the pMCS-AAT-PB:*PGKpuroΔtk* cassette to finalize the targeting construct. The detailed cloning protocol and schematic diagram was represented in Fig. S1b,c.

### Cell culture and gene targeting using CRISPR/Cas9 and PB-based donor plasmid

HeLa-cell derived HIV-1 reporter TZM-bl cells^33^ were maintained in Dulbecco’s Modified Eagle’s Medium supplemented with 10% FBS, 100 U penicillin ml^−1^ and 100 mg streptomycin ml^−1^ at 37 °C and 5% CO_2_. TZM-bl cells (3~5 × 10^5^) were co-transfected with 2 µg of CRISPR/Cas9-gRNA plasmid and 2 µg of targeting vector using lipofectamine^2000^ transfection reagent (Invitrogen, Carlsbad, CA). After 4 days transfection, the cells were selected by puromycin (1 µg/mL) for 2 weeks with daily medium change. Then, single drug-resistant clones were picked and propagated in 12-well plates for further analysis. The primer information can be found in Supplementary Table S1.

### Removal of PB transposon cassette

Two biallelic Cas9-targeted clones were selected for the transposon removal study. To remove the *piggyBac* cassette, TZM-bl cells (3 × 10^5^) with biallelic HR were transfected with 4 µg hyperactive transposase vector, HA-hyperPBase. Seven days after the transfection, immunostaining with CXCR4 specific antibodies (Biolegend) and flow cytometry assay were used to evaluate the efficiency of hyperPBase-mediated CXCR4 reintegration in TZM-bl cells. Synchronously, the single cell clone was picked and propagated for genotyping by PCR analyses with three pairs of primers: P1 and P2 (located in CXCR4 gene), P5 and P6 (paired with *PGK*) and P7 and P8 (*puroΔTK*) to detect the removal of the PB transposon cassette from the genome. These primers are also listed in Supplementary Table S1.

### T7E1 assay for on/off-target analysis

The genomic DNA was extracted from TZM-bl cells transfected with CRISPR-Cas9 and *piggyBac* transposon vector using Blood & Cell Culture DNA Midi Kit (TIANGEN, China) according to the manufacturer’s instructions. PCR was performed for 35 cycles and the genomic region flanking the gRNA target site or potential off-target site was PCR amplified using primers listed in Supplementary Table S2. The fragments were then purified using the Omega gel kit and used for T7 endonuclease 1 assay. 300 ng of PCR product were annealed to form heteroduplexes and digested with T7 Endonuclease I (5 units, New England Biolabs) in a total volume of 20 µl at 37 °C for 30 minutes. The intensities of the bands were analyzed by electrophoresis on a 1.5% agarose gel.

### Flow cytometry analysis

To analyze CXCR4 expression levels or indentify the single clone, the genome-targeted TZM-bl cells were trypsinized into single cells and suspended in PBS for flow cytometry analysis or sorting with phycocrythrin (PE)-conjugated anti-human CXCR4 antibodies. The data were analyzed with FlowJo software (Treestar)

### Quantitative real-time PCR

The relative expression of mRNAs encoding CXCR4 (WT), CXCR4 mutant (MT) were determined by real-time qPCR using SYBR Green PCR Master Mix (Invitrogen). Primers are as follows: CXCR4 (WT) forward: 5′-ATCCCTGCCCTCCTGCTGACTAT-3′ CXCR4 (MT) forward: 5′-TCCCTGCCCTCCTGTTAACTATTC-3′ CXCR4 reverse: 5′-TGGAACACAACCACCCACAAGTC-3′ GAPDH forward: 5′-GAGTCAACGGATTTGGTCGT-3′ GAPDH reverse: 5′-GACAAGCTTCCCGTTCTCAG-3′. GAPDH was used for normalization of the quantitative real-time PCR data. To measure the CXCR4 mutant cells protected against HIV-1 infection, the cellular genomic DNA was isolated for quantitative PCR with primers specific to HIV-1 gag or human β-globin gene. Primers are as follows: gag forward: 5′-ATCAATGAGGAAGCTGCAGAA-3′ gag reverse: 5′-TCCCAGGATTATCCATCTTTTATAG-3′ β-globin forward: 5′-ACACAACTGTGTTCACTAGC-3′ β-globin reverse 5′-TGGTCTCCTTAAACCTGTCTTG-3′. DNA levels were normalized to the cellular housekeeping gene β-globin.

### Western blotting analysis

Total cells were extracted using cell lysis buffer and the lysates were denatured in SDS loading buffer, then the mixture was subjected on SDS-PAGE as described previously^19^. The anti-CXCR4 antibodies (ab2074 Abcam) were used to detect cell surface CXCR4 expression and anti-GAPDH (Cell Signaling Technology) antibodies were served as a loading control.

### HIV-1 infection, luciferase report assay and p24 ELISA

For HIV-1 infection assay, sorted TZM-bl cells (1 × 10^5^) were seeded in a 24-well plate one day before HIV-1 infection. These cells were then challenged with HIV-1_NL4-3_, HIV-1_BH10_, HIV-1_HXB2_ or HIV-1_YU2_ for 8 hours in a total volume of 500 µl serum-free medium. After removing the culture medium and washing cells with PBS 3 times, the cells were cultured in fresh complete media for indicated days. Then, the cells were harvested for luciferase activity analysis with a Bright-GloTM Luciferase Assay System Kit (Promega, WI, USA) by a luminometer (Promega Glomax, USA). At the same time, the supernatant was collected for HIV-1 p24 antigen detection using an ELISA kit (ZeptoMetrix).

### Statistical analysis

All data are presented as the mean ± SD (standard deviation). Statistical analysis was performed using SPSS13.0 for Windows. Comparisons between the two groups were analyzed by paired Student’s t-tests. Comparisons among groups were made by one-way ANOVA test. Differences with P < 0.05 were considered statistically significant. All experiments were repeated at least three times.

## Electronic supplementary material

Supplemental Information
