# Supplementary material for: HIV-1 inhibition in cells with CXCR4 mutant genome created by CRISPR-Cas9 and piggyBac recombinant technologies
Source: Sci Rep. 2018 Jun 5;8:8573. doi: 10.1038/s41598-018-26894-4 (PMC5988798; doi:10.1038/s41598-018-26894-4)
Supplement: Supplementary file 1 — Supplemental Information [file 41598_2018_26894_MOESM1_ESM.pdf]

Supplementary information for

HIV-1 inhibition in cells with CXCR4 mutant genome created by CRISPR-Cas9 and *piggyBac* recombinant technologies

Shuai Liu[1,†], Qiankun Wang[1,†], Xiao Yu[2,†], Yilin Li[1], Yandan Guo[1], Zhepeng Liu[1], Fuyun Sun[1], Wei Hou[1], Chunmei Li[3], Li Wu[4], Deyin Guo[3,*] and Shuliang Chen[1,4,*]

[1]School of Basic Medical Sciences, Wuhan University, Wuhan 430071, P.R. China

[2]Institute of health inspection and testing, Hubei Provincial Center for Disease Control and Prevention, Wuhan 430079, P.R. China

[3]School of Medicine (Shenzhen), Sun Yat-sen University, Guangzhou 510080, P.R. China

[4]Center for Retrovirus Research, Department of Veterinary Biosciences, The Ohio State University, Columbus, OH 43210, USA.

†These authors contributed equally to this work.

*correspondence

Shuliang Chen, PhD, School of Basic Medical Sciences, Wuhan University, Wuhan 430071, P.R. China. Phone: +86-13307101500; Email: Chen-shuliang@whu.edu.cn

Deyin Guo, PhD, School of Medicine (Shenzhen), Sun Yat-sen University, Guangzhou 510080, P.R. China. Phone: +86-20-87335130; Email: guodeyin@mail.sysu.edu.cn

Supplementary information are as follows:

Figure S1. Construction of the *piggyBac* donor vector and sequence analysis.

Figure S2. Flow cytometry analysis of the cell surface CXCR4 expression in different cell lines.

Figure S3. Early apoptosis ratio of different screened cell lines.

Table S1. The primers used in the study

Table S2. The primers used to analyze off-target sites

Figure S1

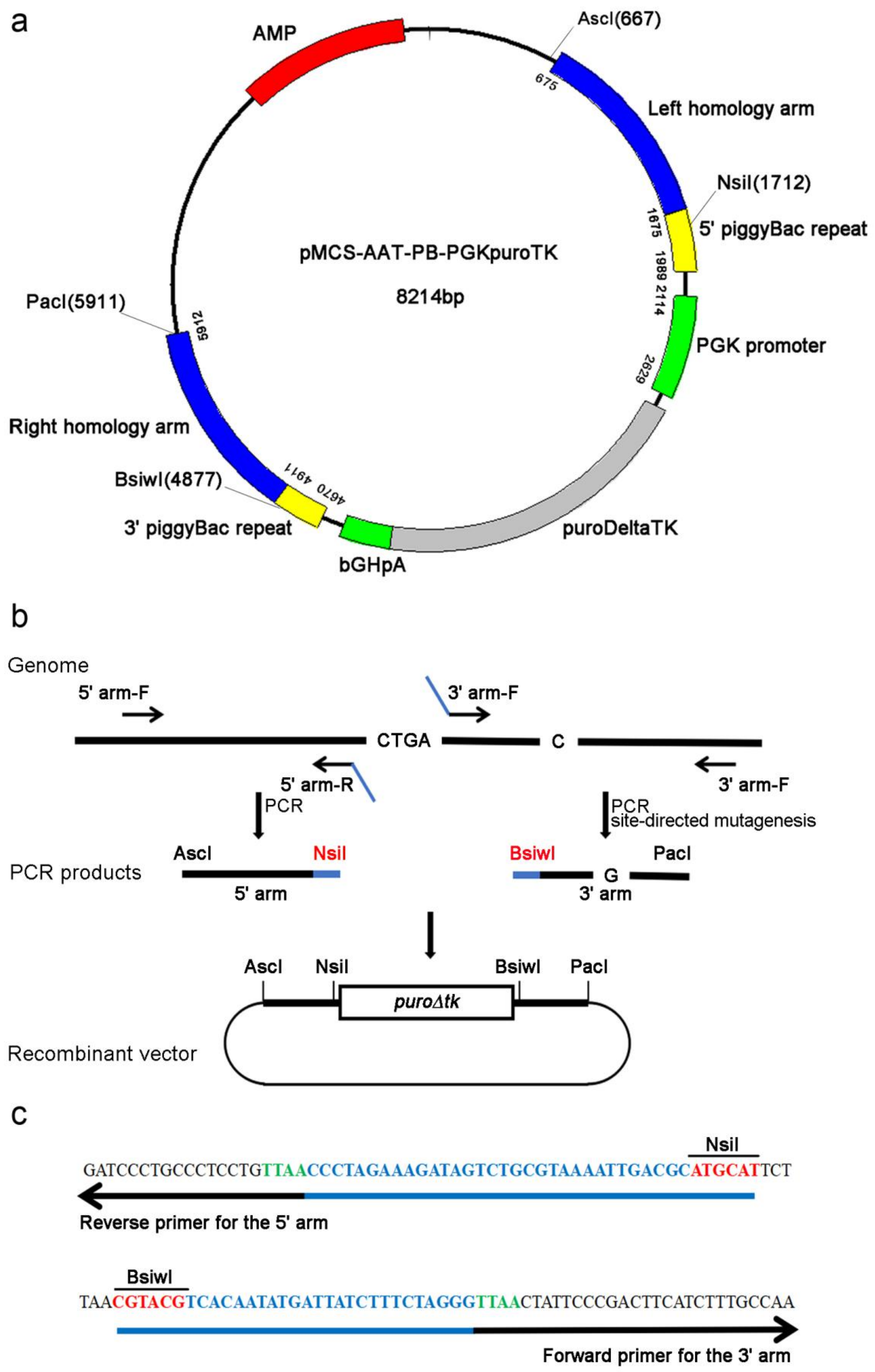

a
AMP
AscI(667)
675
Left homology arm
NsiI(1712)
1675
5' piggyBac repeat
1989 2114
PGK promoter
2629
pMCS-AAT-PB-PGKpuroTK
8214bp
PacI(5911)
5912
Right homology arm
BsiwI(4877)
4911
4670
3' piggyBac repeat
bGHpA
puroDeltaTK
b
Genome
5' arm-F
3' arm-F
CTGA
C
5' arm-R
3' arm-F
PCR
PCR
site-directed mutagenesis
PCR products
AscI
NsiI
5' arm
BsiwI
PacI
G
3' arm
AscI
NsiI
BsiwI
PacI
puroΔtk
Recombinant vector
c
NsiI
GATCCCTGCCCTCCTGTTAACCCTAGAAAGATAGTCTGCGTAAAATTGACGCATGCATTCT
Reverse primer for the 5' arm
BsiwI
TAACGTACGTCACAATATGATTATCTTTCTAGGGTTAACTATTCCCGACTTCATCTTTGCCAA
Forward primer for the 3' arm

Figure S1. Construction of the *piggyBac* donor vector and sequence analysis

(a) Map of *piggyBac* transposon expression plasmid. (b) Schematic of the targeting vector construction. In this example, endogenous C is to be modified to G through PCR and site-directed mutagenesis. The lengths of the homology arms are 1008 bp (5' arm) and 1115 bp (3' arm). Arrows indicate PCR primers; blue portions indicate parts of the transposon sequences and restriction enzyme sites. These homology arms (5' arm and 3' arm) need to be digested by the appropriate restriction enzymes and ligated into the cloning vector together with the transposon fragment. (c) Corresponding sequences of PCR primers adjacent to the transposon for the CXCR4 locus modification. The transposon sequences are shown in blue, the restriction enzyme sites are shown in red, the introduced duplicated TTAA sites are shown in green and genomic sequences are shown in black.

Figure S2

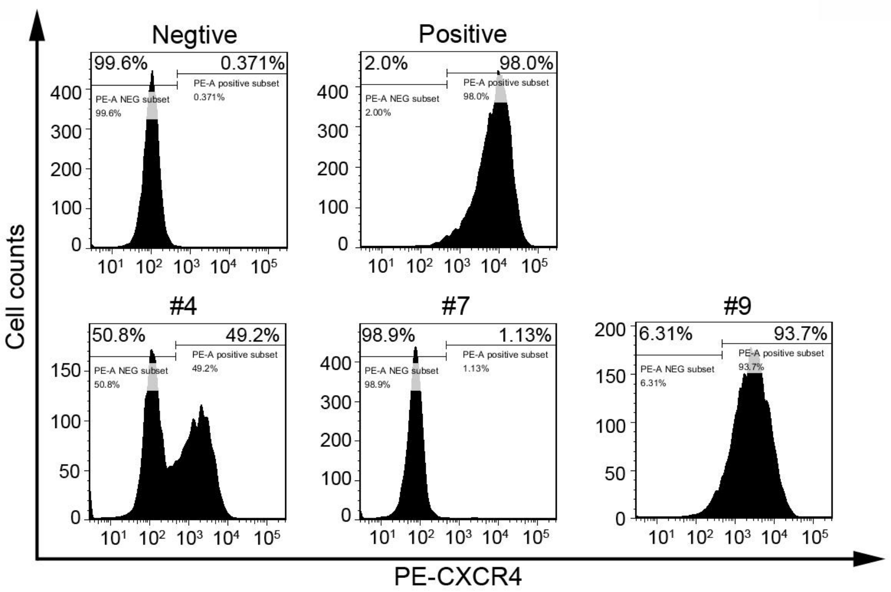

Figure S2. Flow cytometry analysis of the cell surface CXCR4 expression in different cell lines

Flow cytometry to analyze the surface CXCR4 (wild-type and mutant) expression in cells where the transposon was removed from one allele (#4) or the transposon was removed from both alleles (#9). #7 represents a cell clone with transpose integration and without CXCR4 expression. Cells were stained with a CXCR4 antibody (Biolegend) and analyzed by flow cytometry (FACS Ariall, BD). Data are from one representative experiment which was repeated three times.

Figure S3

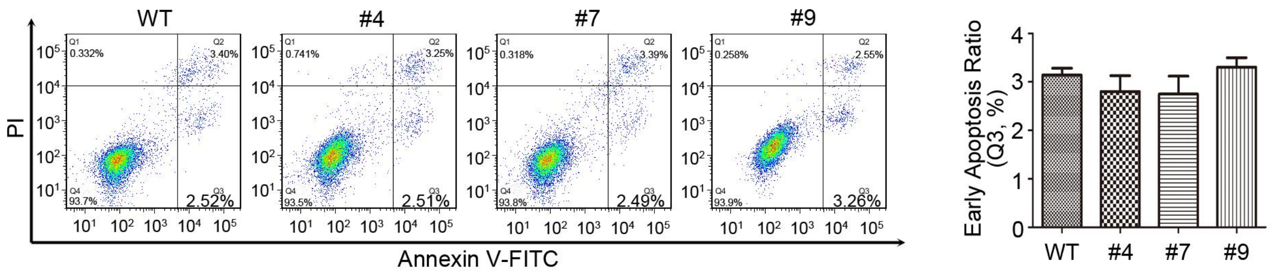

Figure S3. Early apoptosis ratio of the different screened cell lines.

The *CXCR4* editing in different cell lines did not significantly affect cell apoptosis and cell survival. The data shown are the mean ± SD of three independent experiments.

Table S1. The primers used in the study

Name	Primer sequence (5′-3′)
P1	CTACACCGAGGAAATGGGCTCA
P2	TCAAACTCACACCCTTGCTTGAT
P3	GATAAACACGAGGATGGCAAGAGAC
P4	AGATAATCTTTTGACTCACGCGGTC
P5	ATACAGACCGATAAAACACATGCGT
P6	ACTGAGGATACTGGATGAGGAAAGC
P7	TCGAGCGGGTCACCGAGCTGC
P8	GCACCAGGTGCGCGGTCCTTC

Table S2. The primers used to analyze on-target and off-target sites

gRNA	Name	Primer sequence (5′-3′)	locus
/	CXCR4 (783bp)	F:TGGGCTCAGGGGACTATGACTCCATGAAGG<br>R:CAAACTCACACCCTTGCTTGATGATTTCCA	
#1	Off-target 1	F:TTTGGGTTAATGAGTCAATGTGG<br>R:CAGAGCAAGGGTTCACCATTTCC	Chr16:-7232231
	Off-target 2	F:ATGGGAAGGAGTGGTTCTAGGTT<br>R:GGGAGGTAAACTATCCTGGTCAA	Chr9:+116191155
#2	Off-target 1	F：GTCCAGTGGAGCCAATAAAGGCTTG<br>R：TGAGGGTGATTGCTGAGGAGAACA	Chr19:-16625337
	Off-target 2	F:CCAGGGTGAAGGAATGAGGACTG<br>R:GAAGTCGGGATGGTTGGCGTTAT	Chr14:+102482268
	Off-target 3	F:AGCATTCCTGGCGTGGCAAACAC<br>R:GCAGGGCTTAATGGGACAAGTGG	Chr1:-1249145
	Off-target 4	F：TTGGGTTTCCTCCAGGCTGTTAG<br>R：AAGGTCCCATAGCAAGTAGGAGGC	Chr1:-21036229
	Off-target 5	F：CCCTCCACCCACATCCACATTCA<br>R：ATGAGATCTGGCTCCCATTGAAACA	Chr5:-32790650
